# Supplementary material for: Academic motivation, procrastination, and adjustment: Exploring their impact on student profiles and academic performance
Source: PLoS One. 2025 Nov 18;20(11):e0335913. doi: 10.1371/journal.pone.0335913 (PMC12626312; doi:10.1371/journal.pone.0335913)
Supplement: S1 File — (ZIP) [file pone.0335913.s001.ZIP › CodeBook.pdf.pdf]

Codebook

X1_Gender

		Value	Count	Percent
Standard Attributes	Label	Gender		
	Type	Numeric		
Valid Values	0	Male	108	38,0%
	1	Female	176	62,0%

X2_Age

		Value
Standard Attributes	Label	<none>
	Type	Numeric
N	Valid	282
	Missing	2
Central Tendency and Dispersion	Mean	21,21
	Standard Deviation	1,705

Y2_GPA

		Value
Standard Attributes	Label	Y1: Grade point average GPA
	Type	Numeric
N	Valid	232
	Missing	52
Central Tendency and Dispersion	Mean	7,5574
	Standard Deviation	,94721

Year_of_Study

		Value
Standard Attributes	Label	Year of Study
	Type	Numeric

N	Valid	284
	Missing	0
Central Tendency and Dispersion	Mean	6,33
	Standard Deviation	59,127

Final_3_kMean

		Value	Count	Percent
Standard Attributes	Label	Students’ profile		
	Type	Numeric		
Valid Values	1,00	Cluster 1:Highly Motivated and Well-Adjusted Students	76	26,8%
	2,00	Cluster 2: Moderately Motivated and Adjusted Students	107	37,7%
	3,00	Cluster 3: Procrastinated and Poorly Adjusted Students	101	35,6%

PASS_SCORE

		Value
Standard Attributes	Label	PASS Συνολικό Score (Procrastination Assessment Scale for Students· PASS)·
	Type	Numeric
N	Valid	284
	Missing	0
Central Tendency and Dispersion	Mean	12,48
	Standard Deviation	4,698

M1_AMS_EAG_Score

		Value
Standard Attributes	Label	Intrinsic motivation - to know
	Type	Numeric
N	Valid	284
	Missing	0
Central Tendency and Dispersion	Mean	5,0660
	Standard Deviation	1,74787

M2_AMS_EXP_Score

		Value
Standard Attributes	Label	Extrinsic motivation - identified
	Type	Numeric
N	Valid	284
	Missing	0
Central Tendency and Dispersion	Mean	5,3530
	Standard Deviation	1,51719

AMS_ELK_Score

		Value
Standard Attributes	Label	Amotivation
	Type	Numeric
N	Valid	284
	Missing	0
Central Tendency and Dispersion	Mean	1,5977
	Standard Deviation	1,16026

Y1_SACQ_Academic_Adaptation

		Value

Standard Attributes	Label	Student Adaptation to College (SACQ total score)
	Type	Numeric
N	Valid	284
	Missing	0
Central Tendency and Dispersion	Mean	141,3345
	Standard Deviation	23,58004

Frequencies

Statistics

Y1: Grade point average GPA

N	Valid	232
	Missing	52
Mean		7,5574
Median		7,6000
Std. Deviation		,94721
Range		4,59
Minimum		5,00
Maximum		9,59
