# Supplementary material for: Academic motivation, procrastination, and adjustment: Exploring their impact on student profiles and academic performance
Source: PLoS One. 2025 Nov 18;20(11):e0335913. doi: 10.1371/journal.pone.0335913 (PMC12626312; doi:10.1371/journal.pone.0335913)

**Appendix: Cluster Evaluation**

| Method | Best solution must have | KMeans2 | KMeans3 | KMeans4 |
| --- | --- | --- | --- | --- |
| Ball_Hall | max | 1359,511 | 748,7786 | 529,2611 |
| Banfeld_Raftery | min | 2046,082 | **1728,165** | 1856,443 |
| C_index | min | 0,1187007 | 0,07246111 | 0,0530105 |
| Calinski_Harabasz | max | 586,1694 | **698,6341** | 665,9478 |
| Davies_Bouldin | min | 0,5700133 | **0,5653505** | 0,6245814 |
| Dunn | max | 0,0161481 | **0,02139788** | 0,0124277 |
| GDI | max | 0,0161481 | **0,02139788** | 0,0124277 |
| Gamma | max | 0,7377203 | **0,8523075** | 0,8136172 |
| G_plus | min | 0,0655714 | 0,0415815 | 0,0284426 |
| Log_SS_Ratio | min | 0,7317017 | 1,556004 | 2,01295 |
| McClain_Rao | min | 0,3910882 | **0,2625811** | 0,3092421 |
| PBM | max | 9012,936 | **17197,63** | 15293,84 |
| Point_biserial | max | -32,43071 | **-29,69543** | -31,50169 |
| Ratkowsky_Lance | max | 0,2789728 | 0,3068623 | 0,3483649 |
| Ray_Turi | min | 0,1200577 | 0,1460353 | 0,1595468 |
| sd_scat | min | 0,321462 | **0,1221296** | 0,1749533 |
| sd_dis | min | 0,0330813 | **0,01883744** | 0,0451992 |
| S_Dbw | min | 1,256527 | 1,960668 | 2,674839 |
| Silhouette | max | 0,5724178 | **0,5255995** | 0,4882656 |
| Tau | max | 0,5216527 | **0,5434784** | 0,5289521 |
| Trace_W | max | 206148,3 | **384347,8** | 139447 |
| Trace_WiB | max | 2,233695 | **8,778653** | 0 |
| Wemmert_Gancarski | max | 0,6447278 | **0,68486** | 0,6082596 |
| Xie_Beni | min | 163,5773 | 124,4448 | 47,90348 |

Note: The cases where the 3-cluster solution is the most appropriate are shown in The cases where the 3-cluster solution is the most appropriate are shown in **bold**..


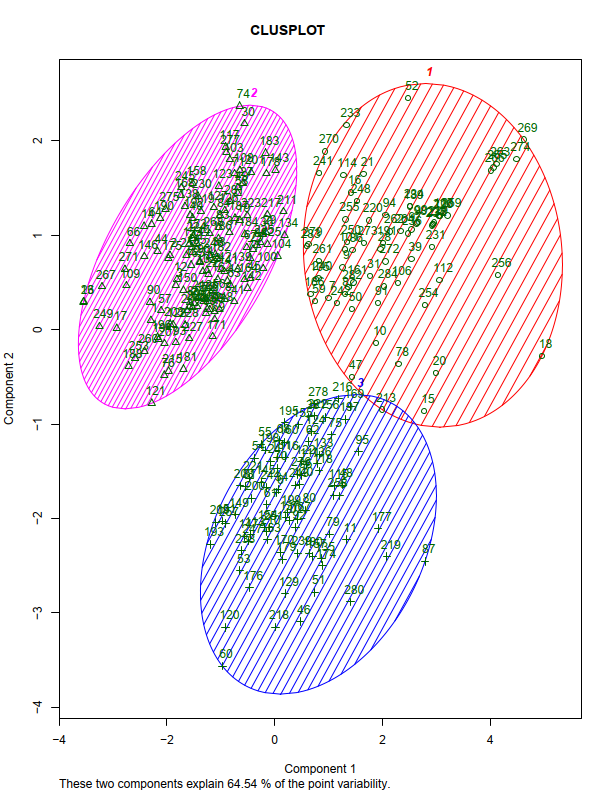

Supplement: S1 File — (ZIP) [file pone.0335913.s001.ZIP › Appendix (Cluster Evaluation).docx.docx]
