# Supplementary material for: Academic motivation, procrastination, and adjustment: Exploring their impact on student profiles and academic performance
Source: PLoS One. 2025 Nov 18;20(11):e0335913. doi: 10.1371/journal.pone.0335913 (PMC12626312; doi:10.1371/journal.pone.0335913)
Supplement: Attachment — Submitted filename: The review result_PONE-D-25-16926.docx [file pone.0335913.s002.docx]

| Clarification on the Study's Context | The article mentions the Greek context for the students, but this could be emphasized further. A deeper exploration of how Greek cultural factors might influence the motivations and adjustment processes could strengthen the relevance of the findings in similar educational contexts. |
| --- | --- |
| Further Justification of Methodology | The authors used K-means cluster analysis and decision trees to identify student profiles. It would be beneficial to expand on why these specific methods were chosen over others. Are there limitations to these methods that could affect the robustness of the results? |
| Implications of the Findings | While the study concludes that intrinsic motivation and academic adjustment are key for academic success, it would be helpful to discuss how these findings can be practically applied. How can universities tailor their academic support services based on these student profiles? |
| Discussion of Limitations | The article briefly mentions the use of self-reported GPA and the cross-sectional design as limitations. However, a more detailed discussion of how these limitations might influence the findings (e.g., biases due to self-reporting or the inability to track long-term trends with cross-sectional data) would be useful. Further, consider whether there are any unaccounted-for variables that might also play a significant role in student adjustment and performance |
| Clarity in Data Presentation | While the statistical analyses are thoroughly presented, some of the figures and tables could benefit from clearer labeling or more detailed explanations. For example, the decision tree model results could be accompanied by a more in-depth interpretation, explaining the decision paths and how they correspond to the student profiles. |
| Suggestions for Future Research | The article briefly mentions future research directions. A more specific outline of how these future studies can build on the current research would be useful. For example, a longitudinal study exploring how these profiles evolve over time could offer deeper insights into how student characteristics and academic success are interconnected |
