# Supplementary material for: Academic motivation, procrastination, and adjustment: Exploring their impact on student profiles and academic performance
Source: PLoS One. 2025 Nov 18;20(11):e0335913. doi: 10.1371/journal.pone.0335913 (PMC12626312; doi:10.1371/journal.pone.0335913)
Supplement: Attachment — Submitted filename: rebuttal letter.docx [file pone.0335913.s003.docx]

**Reviewer #1:** The work presented by these authors is very interesting, but here are some limitations, in my opinion. The introduction is not very engaging, and the authors do not clearly present the limitations of the literature on the paper, nor do they address the gap that their work fills. There is no debate raised regarding the directions of the literature concerning new fields of research on the determinants.

If possible, please consider including a flow diagram for the sample screening process to facilitate better understanding of the sampling procedure. In terms of methodology, the authors do not explain the benefits of using a model and the gaps it addresses, as well as the potential robustness of the results compared to other studies.

**Reviewer #2:** Strengths:

The study employs an appropriate combination of cluster analysis and MANOVA to identify and validate student profiles based on motivation, procrastination, and adjustment.

The table clearly demonstrates meaningful differences between clusters, especially in terms of procrastination and intrinsic motivation.

The profiles generated align with GPA performance, making the results potentially useful for targeted educational interventions.

Suggestions for improvement:Please clarify how the number of clusters was determined (e.g., elbow method, silhouette analysis, or decision tree splits).

**Reply: Thank you for pointing this out, see Appentix**

It would strengthen the findings to elaborate on why extrinsic motivation shows no significant differences across profiles, and whether other subdimensions of motivation were considered.

**Reviewer #3:** The manuscript is generally well-executed, but I have the following suggestions:

Clarify Methodological Choices: It would be helpful to explain in more detail why the K-means and decision tree methods were selected over other potential techniques and the limitations associated with these methods. Contextualization of Findings:

**Reply: Thank you for pointing this out, see Lines 219-232**

Further exploration of how the results relate to the specific cultural and educational context in Greece would strengthen the manuscript, especially considering the unique influences of Greek educational and societal norms on student behavior.

Practical Applications: It would be beneficial to expand on how universities and educational programs can apply these findings in practical terms to support students better, focusing on interventions that target academic procrastination and motivation.

Consideration of Additional Variables: The study focuses on motivation, procrastination, and adjustment. Including additional variables such as socio-economic status or mental health might provide more comprehensive insights into student success.

**Thank you so much for the suggestion, it would indeed be interesting to incorporate these variables in our future studies.**

**Reviewer #4:** Higlights
This research is a great contribution to the field of educational psychology. Although the study focuses on variables that have been extensively studied such as academic adjustment, procrastination, and extrensic and intrinsic motivation, it provides a comprehensive discussion on the dynamic interplay of variables under study in Greeks’ context. Discussion of findings flows logically from the study results.

To enhance the paper for publication, the researchers may consider the following:
1. Provide additional studies in Greece on similar variables to illustrate the study’s context. Only one study in Greece focusing on the positive effect of academic adjustment on students’ academic success was included. It will enrich the study’s context if the researcher will provide additional studies along this topic.

**Reply: Thank you for pointing this out, see Lines 46-56**

2. Revise the introduction to improve its coherence and cohesiveness. The researcher may also include a brief background or context on the status of Greek college students’ academic adjustment, motivation and procrastination in general.
3. Include participants’ relevant demographics such as their year level in college and degree programs because these data can also provide insights to the study’s results.

**Reply: Thank you for pointing this out, see Lines 142-152**

4. Add the title, Results, after Statistical Analysis to provide demarcation between Methodology and Results Sections.

**See line 238**

5. Elaborate on specific interventions recommended in the conclusion section. Would it be school interventions only? Can policy makers be included too?
6. Ensure consistency in writing reference entries, capitalization of journal titles, use of period before the url. Observe hanging indentation.
7. The paper provides interesting, relevant, and insightful findings. To improve its clarity, consider improving awkward sentence construction and cohesion.

**Reviewer #5:** It is good that the manuscript recognizes the need for academia to remain diligent in seeking ways to continuously improve support of student outcomes.

However, in reading the manuscript I struggle to identify the meaningful contribution or expansion of knowledge. The constructs, academic motivation, procrastination, and adjustment have been extensively studied. Many of the findings are readily available and known

Perhaps a more beneficial study could assess the influence of online modalities or select AI policies in relation to academic motivation, procrastination, and adjustment.
