# Supplementary material for: Academic motivation, procrastination, and adjustment: Exploring their impact on student profiles and academic performance
Source: PLoS One. 2025 Nov 18;20(11):e0335913. doi: 10.1371/journal.pone.0335913 (PMC12626312; doi:10.1371/journal.pone.0335913)
Supplement: Attachment — Submitted filename: 2nd rebuttal letter.docx [file pone.0335913.s004.docx]

**Comments to the Author**

6. Review Comments to the Author

Reviewer #1: (No Response)

Reviewer #2: Thank you for the opportunity to review the revised manuscript. The authors have carefully addressed the comments raised in the previous round, and the revisions have significantly improved the overall clarity and quality of the paper. The theoretical framework is now more coherent, the methodology is sound, and the discussion of the findings is insightful and well-supported by the data. I find the manuscript to be well-structured and of potential interest to the journal’s readership. I recommend that the paper be accepted for publication.

Reviewer #3: (No Response)

Reviewer #4: The study is a good addition to existing knowledge in the field of educational psychology that investigates the correlation among variables such as motivation, adjustment to college, and academic achievement.
The author was able to address the reviewers’ recommendations in the revised paper. The introduction incorporates studies in Greece on similar topic as well as research gaps. Ideas in the introduction flow naturally and cohesively. The methodology section is logically sound, clear, and replicable. The author also includes respondents’ relevant demographics and educational implications recommended by the reviewers.

However, I have three recommendations:
1. Include a graphic organizer (e.g. flow chart) for the study procedure from choosing the respondents to the statistical analysis to ma ke it easy to grasp?

**Reply: Thank you for pointing this out, see lines 181-183**

2. Provide a table for the respondents’ demographics to accompany the written description so that at glance, readers can make sense of it.

**Reply: Thank you for pointing this out, see lines 157-161**
3. Add an introductory statement in the Results section before mentioning the findings of the study: As expected, procrastination assessment scale was negatively correlated to intrinsic motivation ...

**Reply: Thank you for pointing this out, see lines 313-318**

Also, take note of the correct usage in the following sentences:
1. Freshmans make up 13.8% of the sample. (Correction: Freshmen)

**Reply: Thank you for pointing this out, see line 141**

2. The sample comprised of 36.2% male and 63.4% female students. (Correction: The sample comprised 36.2% male and ….)

**Reply: Thank you for pointing this out, see lines 138-139**

**Τhe following comments did not require any action from the authors**

1. If the authors have adequately addressed your comments raised in a previous round of review and you feel that this manuscript is now acceptable for publication, you may indicate that here to bypass the “Comments to the Author” section, enter your conflict of interest statement in the “Confidential to Editor” section, and submit your "Accept" recommendation.

Reviewer #1: All comments have been addressed

Reviewer #2: All comments have been addressed

Reviewer #3: (No Response)

Reviewer #4: All comments have been addressed

2. Is the manuscript technically sound, and do the data support the conclusions?

The manuscript must describe a technically sound piece of scientific research with data that supports the conclusions. Experiments must have been conducted rigorously, with appropriate controls, replication, and sample sizes. The conclusions must be drawn appropriately based on the data presented.

Reviewer #1: Yes

Reviewer #2: Yes

Reviewer #3: Yes

Reviewer #4: Yes

3. Has the statistical analysis been performed appropriately and rigorously?

Reviewer #1: Yes

Reviewer #2: Yes

Reviewer #3: Yes

Reviewer #4: Yes

4. Have the authors made all data underlying the findings in their manuscript fully available?

The [PLOS Data policy](http://track.editorialmanager.com/CL0/http:%2F%2Fwww.plosone.org%2Fstatic%2Fpolicies.action%23sharing/1/010f019872deb3d1-164d90c2-01a2-47ff-ba40-604f5d003e06-000000/go-BuoFY7firTGNozXAhjTz5pmqy89IOgodUEywFkAA=222) requires authors to make all data underlying the findings described in their manuscript fully available without restriction, with rare exception (please refer to the Data Availability Statement in the manuscript PDF file). The data should be provided as part of the manuscript or its supporting information, or deposited to a public repository. For example, in addition to summary statistics, the data points behind means, medians and variance measures should be available. If there are restrictions on publicly sharing data—e.g. participant privacy or use of data from a third party—those must be specified.

Reviewer #1: Yes

Reviewer #2: Yes

Reviewer #3: Yes

Reviewer #4: Yes

5. Is the manuscript presented in an intelligible fashion and written in standard English?

PLOS ONE does not copyedit accepted manuscripts, so the language in submitted articles must be clear, correct, and unambiguous. Any typographical or grammatical errors should be corrected at revision, so please note any specific errors here.

Reviewer #1: Yes

Reviewer #2: Yes

Reviewer #3: Yes

Reviewer #4: Yes

7. PLOS authors have the option to publish the peer review history of their article ([what does this mean?](https://track.editorialmanager.com/CL0/https:%2F%2Fjournals.plos.org%2Fplosone%2Fs%2Feditorial-and-peer-review-process%23loc-peer-review-history/1/010f019872deb3d1-164d90c2-01a2-47ff-ba40-604f5d003e06-000000/6YxU5amPG9_BgDloleSFPbzEtUJdZtlT2yhzeWUxYsQ=222)). If published, this will include your full peer review and any attached files.

If you choose “no”, your identity will remain anonymous but your review may still be made public.

**Do you want your identity to be public for this peer review?** For information about this choice, including consent withdrawal, please see our [Privacy Policy](https://track.editorialmanager.com/CL0/https:%2F%2Fwww.plos.org%2Fprivacy-policy/1/010f019872deb3d1-164d90c2-01a2-47ff-ba40-604f5d003e06-000000/5iIvehh18UjA5zw-NMNQNYLoBtRE25boFwW9CUSH1F8=222).

Reviewer #1: No

Reviewer #2: No

Reviewer #3: No

Reviewer #4: No
